# Human-In-The-Loop Control and Task Learning for Pneumatically Actuated Muscle Based Robots

**DOI:** 10.3389/fnbot.2018.00071

**Published:** 2018-11-06

**Authors:** Tatsuya Teramae, Koji Ishihara, Jan Babič, Jun Morimoto, Erhan Oztop

**Affiliations:** ^1^Department of Brain Robot Interface, ATR, CNS, Kyoto, Japan; ^2^Laboratory for Neuromechanics and Biorobotics, Department for Automation, Biocybernetics and Robotics, Jožef Stefan Institute, Ljubljana, Slovenia; ^3^Computer Science Department, Ozyegin University, Istanbul, Turkey

**Keywords:** human in the loop control, pneumatically actuated muscle, biologically inspired multimodal control, human motor learning, electromyography

## Abstract

Pneumatically actuated muscles (PAMs) provide a low cost, lightweight, and high power-to-weight ratio solution for many robotic applications. In addition, the antagonist pair configuration for robotic arms make it open to biologically inspired control approaches. In spite of these advantages, they have not been widely adopted in human-in-the-loop control and learning applications. In this study, we propose a biologically inspired multimodal human-in-the-loop control system for driving a one degree-of-freedom robot, and realize the task of hammering a nail into a wood block under human control. We analyze the human sensorimotor learning in this system through a set of experiments, and show that effective autonomous hammering skill can be readily obtained through the developed human-robot interface. The results indicate that a human-in-the-loop learning setup with anthropomorphically valid multi-modal human-robot interface leads to fast learning, thus can be used to effectively derive autonomous robot skills for ballistic motor tasks that require modulation of impedance.

## Introduction

Human-in-the-loop control systems provide an effective way of obtaining robot skills that can eliminate the need for time consuming controller design (Peternel et al., 2016). Robot self-learning (i.e., reinforcement learning) is another powerful approach for obtaining robot skills; but it usually requires long training unless initialized by a human demonstration (which can be provided easily by human-in-the-loop systems). Conventional controller design is especially problematic for robots with Pneumatically Actuated Muscles (PAMs) due to their intrinsic high non-linearity. Therefore, obtaining controllers by using human-in-the-loop control seems to be a good choice to overcome the modeling difficulties faced in PAM modeling and control. However, how the human *in the loop* would adapt and learn to control the PAM based robots has not been investigated earlier. With this study, to our knowledge, we make the first attempt toward obtaining of a non-trivial skill for a PAM based robot through human-in-the-loop robot control. The motto we adopt in human-in-the-loop robot control is “let us utilize human brain to do the learning and optimization for control.” Note that we make a distinction between human-in-the-loop control and kinesthetic teaching based studies (Hersch et al., 2008; Kronander and Billard, 2014; Tykal et al., 2016), as in the former human is the learning controller generating motor commands in real-time as opposed to being an active scaffold or a guide to the robot. After skilled operation is achieved by the human, autonomous controller synthesis boils down to mimicking human behavior by the help of a computer as a function of state and/or time and sometimes context. To ensure a smooth integration of the human into the control loop, the interface between the robot and the human operator is critical. The interface often necessitates anthropomorphic human-robot mapping with intuitive mechanisms to engage the sensorimotor system -as opposed to the cognitive system- of the human operator. Such an interface makes it possible for the human to learn to control the robot and do useful tasks with it as a tool in short timescales. In recent years, there has been a growing interest in human-in-the-loop robotic systems for robot skill synthesis (e.g., Walker et al., 2010; Babic et al., 2011; Ajoudani et al., 2012; Moore and Oztop, 2012; Peternel et al., 2014). However, with a few exceptions [e.g., Ajoudani et al. (2012) who used human muscular activity from antagonistic pairs for end-point impedance estimation in teleoperation, and Walker et al. (2010) who proposed a system utilizing a hand grip force sensor to modulate the impedance of the robot during the teleoperation], the majority of the existing studies are targeted for position control based tasks. In Peternel et al. (2014), the authors have shown that human sensorimotor system could drive a robot using multimodal control. In this work, in addition to the usual position based teleoperation, hand flexion was measured by muscle electromyography (EMG) and used to set the compliance property of the robot in real-time. Although the interface was intuitive, the human operator had to perform an additional task of squeezing a sponge ball to create muscle contraction to deliver the required EMG signals to regulate the stiffness of the robot. A more direct control system can be envisioned for those robots that have antagonistically organized muscle actuation system akin to biological systems. Such robot architectures can be built by using so-called artificial muscles, e.g., by Pneumatically Actuated Muscles (PAMs). In such a case, the human muscle activities can be measured in real-time and channeled to the corresponding artificial muscles of the robot in an anthropomorphically valid way (i.e., biceps to “robot biceps;” triceps to “robot triceps”). However, driving a robot with control signals based purely on muscle activities is not trivial if not impossible due to factors such as noise in acquisition, motion artifacts, and the differences in the muscle organization of the robot and the human.

With this mindset, we propose a multimodal approach to control a Pneumatically Actuated Muscle (PAM) based robot where EMG signals and the elbow angle of the human arm are anthropomorphically mapped to the robot creating an intuitive control scheme. The proposed approach is realized on a simple single joint robot, and autonomous behavior of hammering a nail into a wood block is synthesized through human sensorimotor learning. Subsequently, a set of experiments is conducted for analyzing human adaptation to the developed human-in-the-loop control setup. The results indicate that such a system can be adopted to effectively derive autonomous controllers for ballistic motor tasks (Brooks, 1983). In addition, to show the usefulness of our approach to design controllers for a non-linear robot system that is difficult to model, we compared the autonomous controller acquired through our human-in-the-loop system and the controller derived by a model-based optimal control method.

## Methods

**Figure 3:**
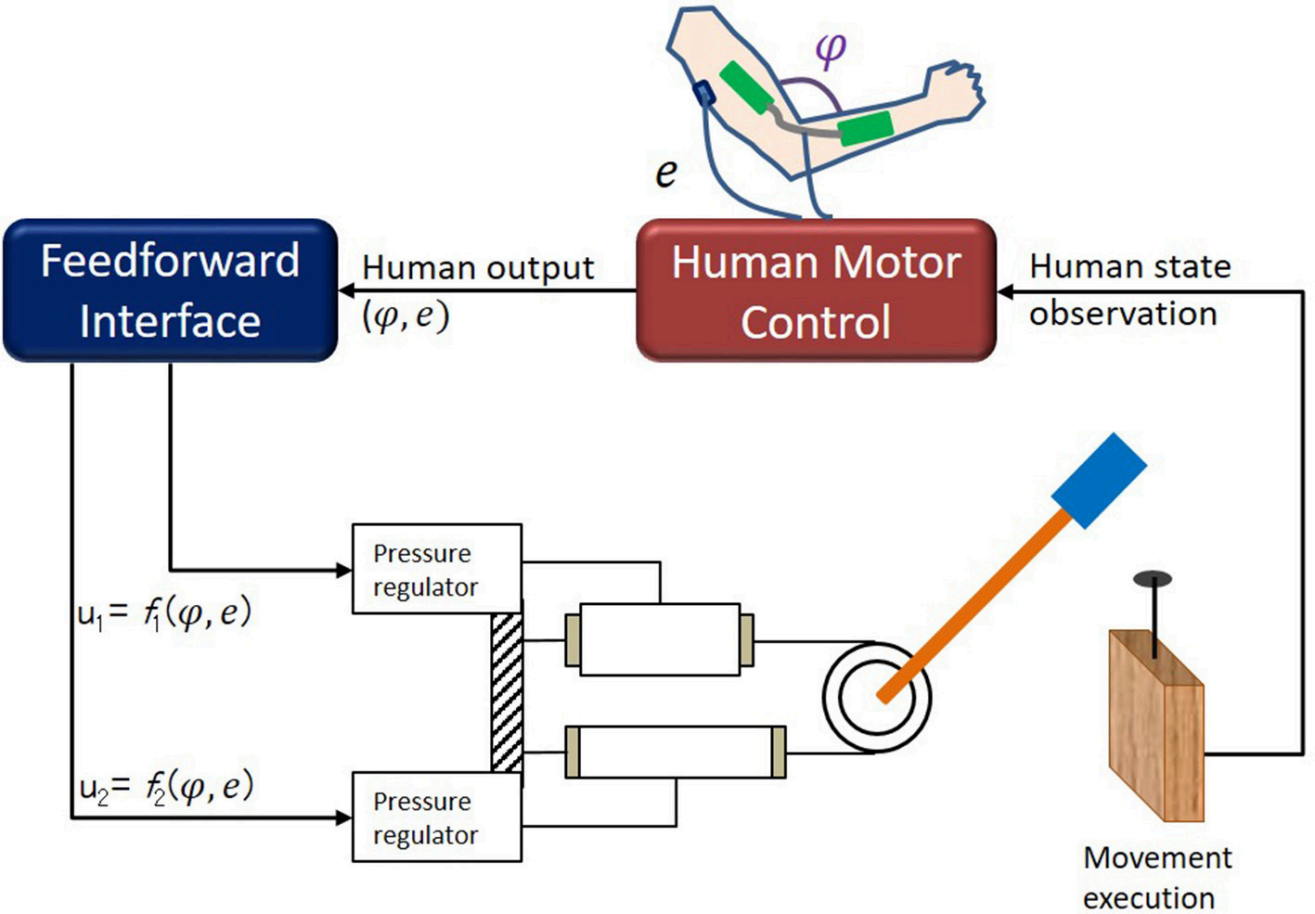
Hammering setup. Wood block was vertically placed, and had 9 cm thickness. We used nail of 5 and 0.23 cm thickness. Hammering task was initialized by inserting nail into wood by ~0.4 cm and placing nail under center of plastic end-effector attachment that served as hammer head.

One of the factors driving this study is to investigate how human-in-the-loop robot learning can be naturally generalized to tasks that go beyond position control. In particular, we aim at generating autonomous skills based on force based policies. To realize this as a proof of concept we start from a simple one joint two degrees-of-freedom Pneumatically Actuated Muscle (PAM) based robot that has an antagonistic actuation design allowing the stiffness of the robot to be controlled through co-activation. The general framework realizes an anthropomorphic mapping for human to control the robot in real-time by using arm movements and muscle electromyography (EMG) signals from the arm so that the position and stiffness control can be achieved simultaneously. Once this is achieved then various tasks where the robot must change its stiffness for successful execution can be given to the control of human operators for shared control (Dragan and Srinivasa, 2013; Amirshirzad et al., 2016) or autonomous skill synthesis (Babic et al., 2011; Moore and Oztop, 2012; Peternel et al., 2014) purposes. The framework is illustrated in Figure 3 in the special case of nail hammering task. How the EMG signals and the human movements are converted to PAM pressures is left for the designer. In a classical setting, it may include torque-to-pressure feedforward model as part of the human-robot interface; but, we favor a more direct approach to offload this mapping to human sensorimotor system to be learned as the part of task execution.

### Hardware setup

**Figure 1:**
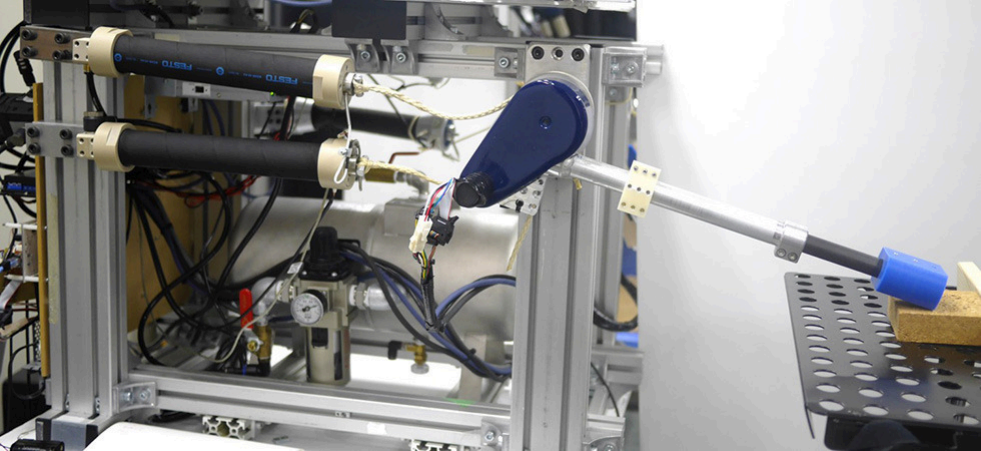
One joint robot is composed of antagonistically organized Festo MAS-40 pneumatic artificial muscle (PAM) pair. Each PAM is connected to rotational disk/pulley system by string tendons housing arm of 35 cm. Pressurizing PAMs creates opposing torques on disk, therefore it is possible control both the motion and stiffness of arm through pressure control. Hardware consists of load cells between tendon and muscle-ends that can be used for control.

The one joint robot is composed of an antagonistically organized Festo MAS-40 pneumatic artificial muscle (PAM) pair (see Figure 1) (Noda et al., 2013; Teramae et al., 2013). Each PAM is connected to a rotational disk/pulley system by string tendons housing an arm of 35 cm. Pressurizing the PAMs creates opposing torques on the disk, therefore it is possible the control both the motion and stiffness of the arm through pressure control. The hardware consists of load cells between the tendon and muscle-ends that can be used for control. A feed-forward model representing the relation between air pressure and the resulting muscle/torque can be learned or derived (Ching-Ping and Hannaford, 1996) to control the muscles and the robotic system that it belongs. Due to highly non-linear relations between system parameters it is considered difficult to control such systems. In the current study, as human was placed in the control loop, we eliminated the torque-pressure modeling and leave it for human operator to learn it as a part of task execution. As described below, human was given a simple interface to directly control the pressures in the PAMs to achieve the task at hand.

**Figure 2:**
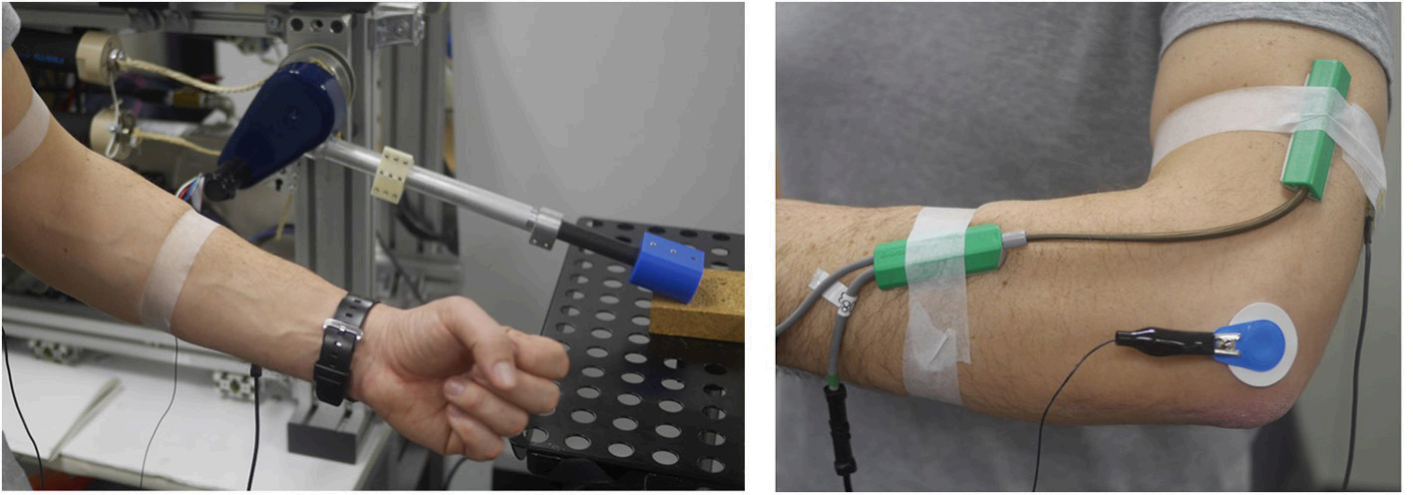
Surface EMG was used to measure muscle activities and digital goniometer (Goniometer SG150, Biometrics Ltd.) was used to measure human elbow angle. Interface program we developed used these signals in real-time to generate desired pressure values for PAM of robot at 250 Hz. EMG electrodes was attached to skin over triceps muscle for hammering task.

A digital goniometer (Goniometer SG150, Biometrics Ltd.) was used to measure the human elbow angle, and surface EMG was used to measure muscle activities (see Figure 2). The EMG signals were used in real-time to generate desired pressure values (**u**) for the PAM of the robot at 250 Hz. The desired pressure values were realized by a proportional valve controller (provided by NORGREN). The EMG electrodes were attached to the skin over the triceps muscles. EMG signals were passed through rectification and low pass filtering.

### Human-robot interface

A generic interface to output the desired pressure values to the PAMs can be given with **u** = **W**[1 φ *e*]^*T*^ where **u** is the vector of desired pressures for the PAMs; φ is the elbow angle of the human, and *e* indicates the muscle activity levels. The constant 1, enables a pressure bias to be given to PAMs. In short, **W** is a linear coefficient matrix that maps EMG and joint movement data of the human directly to PAM (desired) pressures and is composed of bias terms (*B*_*U*_, *B*_*L*_), positional factor (*K*^φ^) and EMG factor (*K*^*e*^). A non-linear mapping could have been used; but, as we would like to rely on human ability to learn to generate appropriate control signals, simplest possible mapping, i.e., linear, was deemed appropriate.

To allow ballistic explosive movements that are necessary for hammering, we designed the **W** matrix by inspiring from biology: we created reciprocal inhibition mechanism between the human arm and the robot. To be concrete, the human triceps EMG signal was channeled to the upper PAM (akin to biceps) as an inhibitory signal. The neural control of movement in the human follows a similar design: when the triceps are activated for arm extension, an inhibition signal is sent to the biceps for reducing the effective stiffness of the arm which enables high velocity movements (Ching-Ping and Hannaford, 1996). Since the hammering task relied on extension of the arm for impact, we did not use EMGs from the biceps in this task for experimental convenience. The lower PAM on the other hand was controlled by the human arm angle measured via a goniometer. Overall, the explained feedforward interface was specified with

(1) $$\begin{array}{r} \text{W}=\left[\begin{array}{ccc} B_{U} & 0 & K^{e}\\ B_{L} & K^{\varphi } & 0 \end{array}\right]. \end{array}$$

**Figure 4:**
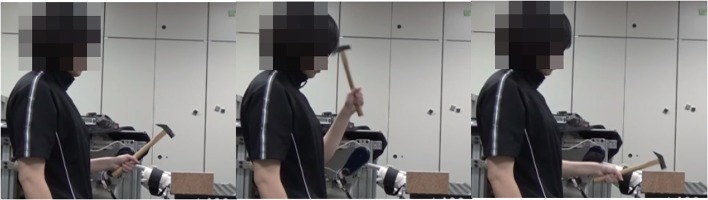
Calibration phase for human-robot interface: We measure minimum and maximum angle and maximum EMG signals while actual hammering task with real hammer and fit the parameters of (1) based on measured data. We obtained informed consent for the publication of this figure from the participant.

**Figure 5:**
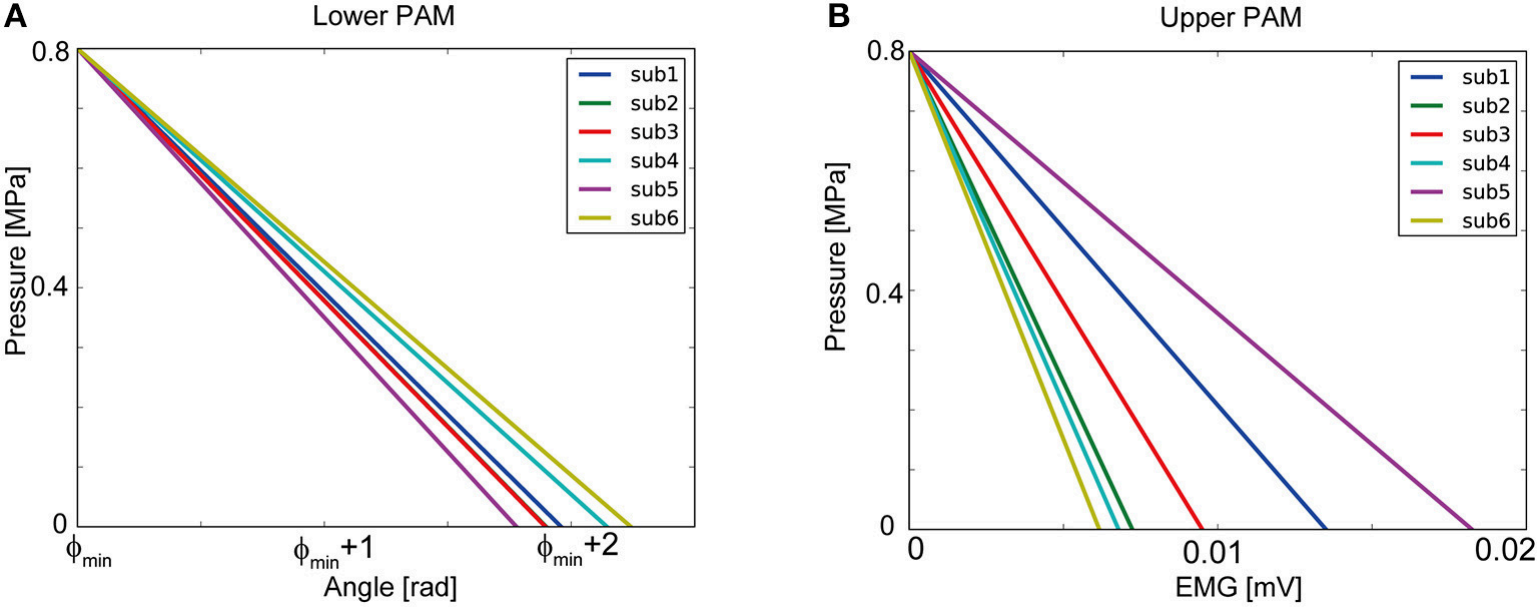
Our control interface for each participant: **(A)** shows relationship of lower PAM pressure controlled and elbow joint angle. **(B)** shows relationship of upper PAM pressure and EMG of triceps.

The parameters that linearly map the goniometer read angles to lower PAM pressure was obtained for each participant through a simple calibration procedure to cover the allowed range of pressure. The parameters for mapping the EMG signals to upper PAM was obtained in a similar fashion. These parameters were kept fixed through the nailing experiments reported in this article. In sum, after the calibration we ended up with formulae weight matrix to map human actions to desired pressures for each participant. Concretely, each participant was asked to conduct hammering movements as depicted in Figure 4. We measured the elbow joint angle and triceps EMG during the movements. From the measured data, maximum (φ_max_), minimum (φ_min_) joint angles and the maximum triceps EMG amplitude (*e*_max_) were identified for each participant. These variables were utilized to derive the interface parameters in Eq. (1) so that minimum and maximum joint angles were mapped to maximum (*P*_max_ = 0.8 [*MPa*]) and minimum (*P*_min_ = 0 [MPa]) desired pressure for lower PAM as depicted in Figure 5A:

(2) $$\begin{array}{rcl} K^{\varphi } & = & -\frac{P_{\max}}{\varphi_{\max}-\varphi_{\min}},\\ B_{L} & = & {\left(1+\frac{\varphi_{\min}}{\varphi_{\max}-\varphi_{\min}}\right)P}_{\max}. \end{array}$$

Similarly, the maximum EMG amplitude of each participant during the real hammering movement was mapped to the maximum (*P*_max_ = 0.8 [*MPa*]) desired pressure of upper PAM as depicted in Figure 5B:

(3) $$\begin{array}{rcl} K^{e} & = & -\frac{P_{\max}}{e_{\max}},\\ B_{U} & = & P_{\max}. \end{array}$$

It is worth underlining that the goal of human movement-to-robot control input mapping is not to make the robot imitate the human exactly; the critical requirement is to obtain an intuitive control by having users see a consistent near real-time response from the robot.

## Experiments

### Experimental design

For the hammering task the robot tip was attached a hard plastic to serve as the hammer head. A compressed wood was used as the material the nail needed to be driven in. Figure 3 illustrates the hammering set up schematically. The wood block was vertically placed, and had 9 cm thickness. We used a nail of 5 and 0.23 cm thickness. The hammering task was initialized by inserting the nail into the wood by ~0.4 cm and placing the nail under the center of the plastic end-effector attachment that served as the hammer head. Experimenter detect the task termination when the nail could be completely driven into the wood.

The experiments were designed as a series of sessions in which several trials of human-in-the-loop robot control for driving the nail into the wood was run. Each trial consisted of 15 s of robot teleoperation in which the participants executed hammering movements in real-time via the robot. Participants were shown that their arm movement was imitated by the robot, and a muscle contraction caused movement on the robot even though their arm was still. Furthermore, participants were given the freedom to hammer the nail as they like so the frequency of the strikes (hammering motion) and the amplitude of the robot motion varied from participant to participant. Each session deemed to be complete when the nail could be completely driven into the wood. Then the nail was reset to its default position (care was taken to place the nail in a fresh new location on the wood block). As a measure of performance, we took the number of trials, i.e., the number of 15 s blocks that it took the participant to drive the nail into the wood. We allowed a maximum of 5 trials for each session. The experimental data showed that this was sufficient for driving the nail into the wood for even novice participants.

To summarize, in the experiments, each participant went through 4 sessions. Each session took a maximum of 5 trials, where each trial was a fixed 15 s robot teleoperation. The number of strikes that a trial contained was up to the participant. Likewise, the number of trials that a session included was dependent on how successfully the participant could hammer the nail, thus varied among participants and sessions.

### Skill transfer with direct imitation (policy copying)

Once a participant learns to drive the nail into the wood, his/her task execution data can be used to construct an autonomous controller. One of the good performing participants was selected for autonomous skill generation. Furthermore, we selected the desired pressure sequences for the lower and the upper PAM control that generated the highest impact among the hammering movements of the selected participant. Since the velocity is proportional to the impact force, we estimated the impact force from the tip velocity of the robot. The human generated pressure trajectories were segmented by taking the moment of upper PAM pressure rise as the *start*, and by taking the moment of collision with the nail as the *end*. For autonomous execution, the obtained pressure trajectories were then reproduced on the robot in a cyclic manner during an execution session (e.g., 15 s).

### Optimal control solution

To compare our model-free human-in-the-loop approach with a model-based controller, we design a policy based on an optimal control method as explained below.

Let *U*_1_≡{*u*_1_, *u*_2_, ⋯ , *u*_*N*−1_} be a sequence of control variables *u*∈ℝ and denote state variables *x*∈ℝ, optimal state and control trajectories are derived by solving an optimal control problem under non-linear system dynamics:

(4) $$\begin{array}{l} \underset{{\text{U}}_{1}}{\text{min}}\text{ J}\left(x_{1},U_{1}\right),\\ s.t.\;x_{t+1}=f\left(x_{t},u_{t}\right). \end{array}$$

where the objective function of the total cost *J*(**x**_**1**_, **U**_**1**_) is defined as being composed of the terminal cost function *l*_*f*_(*x*) alone:

(5) $$J\left(x_{1},U_{1}\right)=l_{f}\left(x_{N}\right).$$

The state and control variables consisted of $\text{x}={\left[\theta ,\overset{\cdot }{\theta },{\text{P}}_{\text{U}},{\text{P}}_{\text{L}}\right]}^{⊤}$ and $\text{u}={\left[\tau_{u},\tau_{l}\right]}^{⊤}$, respectively. *P*_*U*_ and *P*_*L*_ are air pressures of the upper and lower PAMs. In this case, we considered a cost function model,

(6) $$\begin{array}{r} {\text{l}}_{f}={w_{1}{\left(\theta \left(T\right)-\theta_{ref}\left(T\right)\right)}^{2}+w}_{2}{\left(\overset{.}{\theta }\left(T\right)-{\overset{.}{\theta }}_{ref}\left(T\right)\right)}^{2}, \end{array}$$

where θ_ref_(*T*) and ${\overset{\cdot }{\theta }\;}_{\text{ref}}\left(T\right)$ are a target terminal joint angle and target terminal angular velocity obtained from the strongest hammering trajectory of the selected participant. Weights of *w*_1_ and *w*_2_ were optimized by Inverse optimal control (IOC) framework with the learned hammering data of one participant (see Appendix).

To solve the optimal control problem, we derived dynamics model of the 1-DoF robot,

(7) $$\begin{array}{r} I\ddot{\theta \;}+h\left(\overset{\cdot }{\theta }\right)+g\left(\theta \right)=\tau^{u}+\tau^{l}, \end{array}$$

where the inertial parameter is represented as I. The term $h\left(\overset{\cdot }{\theta }\right)$ stands for the friction model:

(8) $$\begin{array}{r} h\left(\overset{\cdot }{\theta }\right)=D\overset{\cdot }{\theta }+\Gamma_{1}\tanh\left(\Gamma_{2}\overset{\cdot }{\theta }\right), \end{array}$$

which is composed of viscous and static friction models. *D* is the parameter of the viscous friction. Γ_1_and Γ_2_ are the static friction parameters, and *g*(θ) represents the gravity term. τ^*u*^ and τ^*l*^are torques generated by the upper and lower PAMs, respectively. The torque was calculated with a model of a PAM actuator as in Teramae et al. (2013). We convert the continuous time robot dynamics Equation (7) to a discrete time model to formulate the optimal control problem described in Equation (4). We applied an optimal control method, namely iterative Linear Quadratic Gaussian (iLQG) (Todorov and Li, 2005) to obtain the control inputs for executing the nailing task with the robot.

## Results

### Human control adaptation and learning

**Figure 6:**
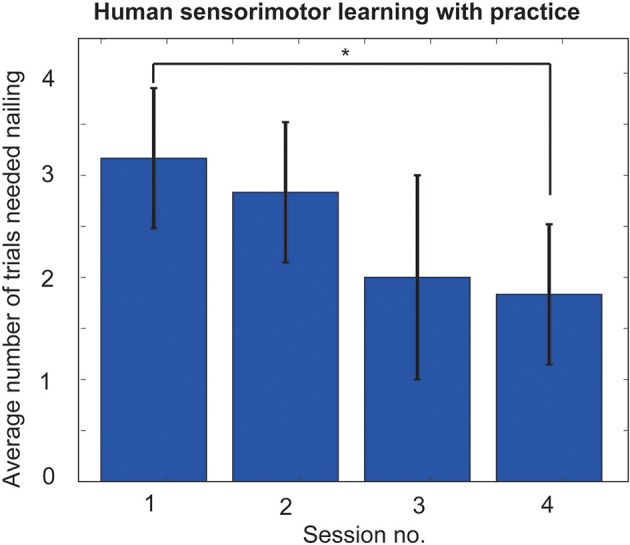
Learning performances of “hammering with robot” experiments. After first session most participants were able to generate occasional high impact strikes; however it took more time for hammering behavior to stabilize into a regular pattern. Hammering performance were much improved after four training sessions (*p < 0.01).

Six participants participated in “hammering with robot” experiments. All the participants showed clear learning effects. After the first session most participants were able to generate occasional high impact strikes; however it took more time for hammering behavior to stabilize into a regular pattern. As presented in Figure 6, the hammering performances of the participants improved, i.e., they could drive the nail with less number of strikes as they become more experienced with the system. A *t*-test comparing the first and last session performances showed that there was a significant improvement in the performance of the participants from the first session to the last (*p* < 0.01), indicating significant human learning.

### Autonomous hammering with direct imitation (policy copying)

**Figure 7:**
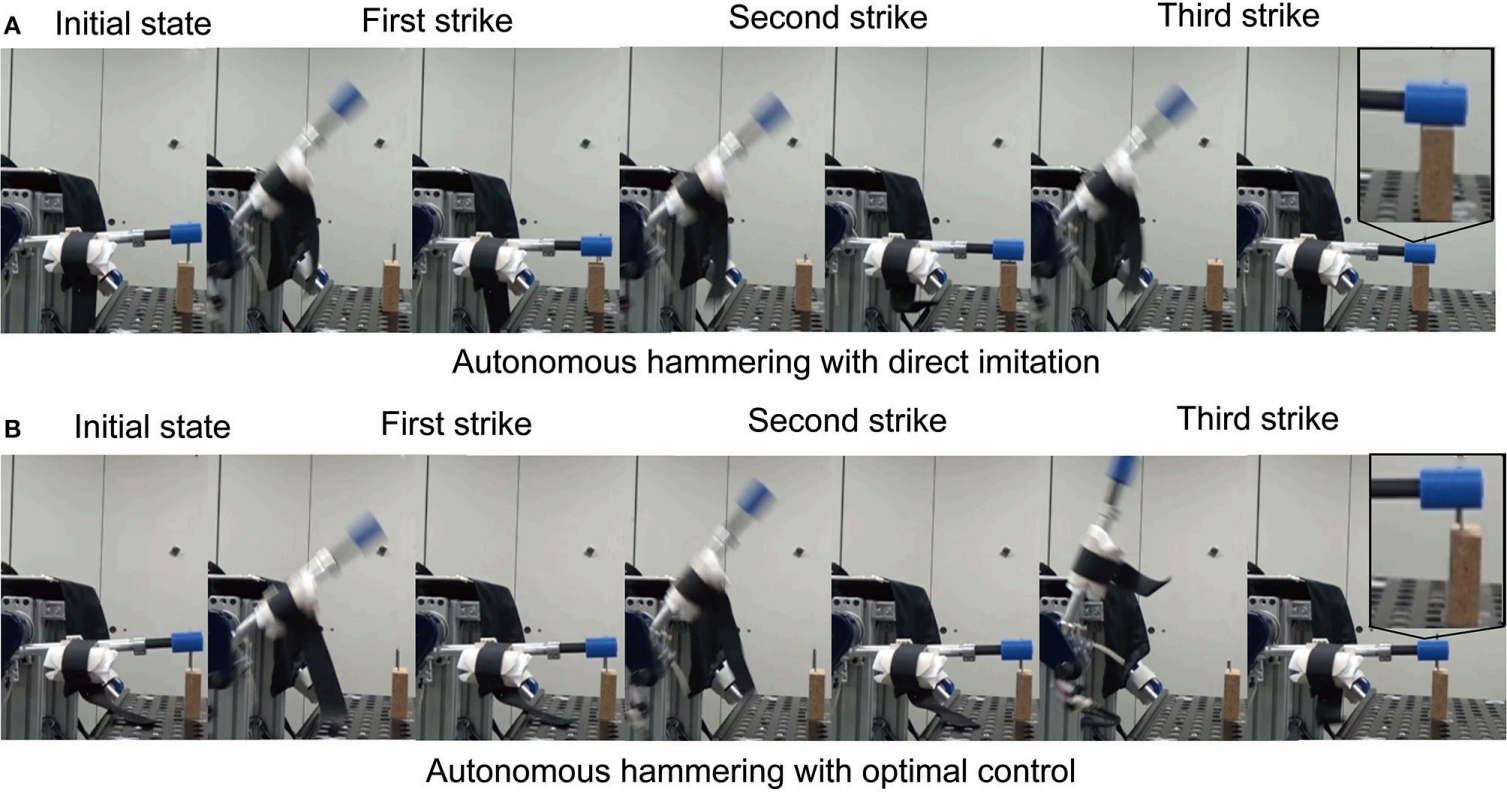
Several video frames illustrating autonomous nail hammering. **(A)** shows autonomous hammering with direct imitation. **(B)** shows autonomous hammering with optimal control.

We selected strongest hammering data from high performance participant. In this case, strongest hammering means hammering with the fastest swing down speed, since the impact force is proportional to the swing down speed. We allowed 15 s of autonomous execution. Figure 1 shows sample frames from an autonomous hammering with direct imitation. The obtained controller could nail with only 3 strikes (Figure 7A). Also, direct imitation of other participants can achieve the nailing (Table 1). As a stress test, we switched to a larger nail of 6.5 cm length and 0.34 cm thickness, and applied the autonomous controller obtained with the original nail (0.23 cm thick and 5 cm long) to the larger nail. The robot could also completely drive this nail, albeit now with 5 strikes.

**Table 1:** Number of strikes required to accomplish the hammering task by autonomous hammering with direct imitation from 6 participants.

**Participant**	**Participant 1**	**Participant 2**	**Participant 3**	**Participant 4**	**Participant 5**	**Participant 6**
Strike count	3	20	25	17	6	8

### Comparison with the policy derived by an optimal control method

To optimize the trajectory and pressure input by using optimal control method, we set the terminal angle and angular velocity based on the selected high impact hammering trajectory. We derived weights of objective function by IOC: we extracted 6 strikes form the final session data of the high performing participant to form the learning data for IOC. As a result, the weights of *w*_1_ = 72.45 and *w*_2_ = 0.033 were obtained. The optimal input and trajectory to be used in execution were then obtained by an optimal control method with the obtained objective function. We allowed 15 s × 5 trials of autonomous execution. Figure 7B shows some sample frames from an autonomous hammering session that employed the trajectories obtained by the optimal control method. The obtained controller could not completely nail within 5 trials (i.e., 40 strikes). These results clearly show the advantage of using our human-in-the-loop approach to derive controllers for non-linear robot systems that is difficult to be identified.

## Discussion

One of the bottlenecks for the introduction of multipurpose robots to human life is the necessity of programming them. It is not feasible to preprogram them for all possible task scenarios. Many methods such as visual demonstration (Pillai et al., 2015), haptic guidance (Power et al., 2015), motor primitive (Peter and Schaal, 2008), and optimization control based (Zhang et al., 2015) methods have been proposed for acquiring robot skills. However, most methods are geared toward systems in which position and force can be reliably controlled. For such systems, conventional methods may deliver suitable solutions for skilled robot behaviors. However, for those systems where position and force control is problematics as in PAMs, it is not effective to use model-based optimization and/or skill transfer methods based on kinematics and force. Needless to say, some studies do exist addressing the precise control of position and force in PAMs (Ching-Ping and Hannaford, 1996; Ugurlu et al., 2015), which nevertheless, have some drawbacks due to the need for complex calibration.

Teaching by demonstration framework is an effective way to rapidly synthesize skills on a robot, when the interface and modality of control is natural for the demonstrator. There are several variants as to how teaching is done from visual demonstration (Dillmann, 2004) to kinesthetic guidance (Calinon et al., 2001; Kushida et al., 2001). In the latter case, the actions are already realized on the robot so no complex processing is needed to reproduce it on the robot. In the former case, even special tracking sensors are used, significant effort may be needed to map the demonstrated movement into robot actions (Ude et al., 2010). These methods, however, may not be always suitable when the targeted task involves non-negligible dynamics and/or fast actions are required. Of course, it is possible and thus often the case that these methods are used to generate initial robot policies that are subject to optimization or improvement via reinforcement learning (Kober et al., 2012). In what we call robot skill synthesis through human-in-the-loop control and learning, we aim to engage the *human sensorimotor system* to do the learning and optimization. Therefore, we seek interfaces and adaptive mechanism for the robot to speed up human learning and minimize the mental and physical effort of the human. In particular, exploiting anthropomorphic similarity of the robot and human (Moore and Oztop, 2012; Oztop et al., 2015), simultaneous human-robot learning (Peternel and Babic, 2013; Mohammad and Oztop, 2015), control mixing and intention understanding (Dragan and Srinivasa, 2013; Amirshirzad et al., 2016) seem to be promising directions to pursue for highly effective human-in-the-loop control systems. As a final note, PAM based robots can be suitable for exploiting human sensorimotor learning effectively as there are parallels with human skeleto-motor system and those robots that employ PAMs with antagonistic setups. Therefore, it seems reasonable to target more complex tasks on higher degrees of freedom robots with PAMs.

## Conclusion

In this study, we proposed and realized a biologically valid multimodal human-in-the-loop system on an antagonistically designed pneumatically actuated one link, two artificial muscled robot. We focused on the ballistic movement of hammering a nail into a wood block, and ran experiments to assess the learning progress of humans to use the robot for driving a nail into a wood block. The rapid human adaption and learning observed, suggest that the developed system engages human sensorimotor learning and does not incur much burden for the cognitive system. In addition to the human experiments, we used one of the high performing participant's skilled execution of the task to synthesize an autonomous controller. The experiments with the controller showed that a significantly larger nail (0.34 cm thick, 6.5 cm long) compared the original one (0.23 cm thick, 5 cm long) used in the skill transfer can be handled with a fixed set of parameters over the conditions. Overall, the current study suggests that adoption of human-in-the-loop approaches for PAM based robots is a fruitful research direction, in which easy and intuitive human learning facilitate effective skill transfer for tasks that require continuous modulation of impedance.

## Author contributions

TT worked mainly in experiment and wrote related sections. KI worked in experiment and wrote related sections. JB supported to improve the paper quality. JM supported about experimental protocol and improved the paper quality. EO worked in experiment and wrote the paper.

### Conflict of interest statement

The authors declare that the research was conducted in the absence of any commercial or financial relationships that could be construed as a potential conflict of interest.

## Appendix

### Optimization of objective function with IOC

To determine the weights of objective function for the optimal control method, we used an inverse optimal control (IOC) method. IOC can estimate the reasonable weights to match the optimal states to the demonstrated behaviors. Then we adopted a probabilistic local IOC approach (Levine and Koltun, 2012), in which the probability of the actions is approximated locally around expert's demonstrations (Park and Levine, 2013). In the local IOC approach, given example trajectories $D=\{X_{1},X_{2},\cdots \}$, the expert's behaviors are represented with a probabilistic model:

(A1) $$p\left(D|l\right)=\prod_{i}^{}p\left(X_{i}|l\left(w\right)\right).$$

After applying Laplace approximation to the model, the weights **w** are learned by maximizing its likelihood. We used the six hammering behaviors of the selected good performing participant to find the parameters of the objective function *w*_1_ and *w*_2_ in Eq. (6).
